# Andean Body: Plastic Surgery With a Cultural Perspective

**DOI:** 10.1093/asjof/ojaf025

**Published:** 2025-04-25

**Authors:** Raúl Martín Manzaneda Cipriani, Hassan Ben Moussa, Stefan Danilla, Zamir Eid Páez Mojica, Aldo Renso Ronquillo Soxo, Edwin Zara León, Juan Manuel Meder Rio, Edgar Sandoval Guzmán, Facundo José Livio

## Abstract

The authors of this study propose the identification and description of the physical characteristics of the Andean body. A person with an Andean body is considered to come from the Andes and areas with higher altitude, which cross areas of Peru, Argentina, Chile, Bolivia, Ecuador, Colombia, and western Venezuela. The objective of the authors of this study is to define the physical characteristics of the Andean body identified in a sample of 1500 female patients. The authors analyzed the physical characteristics of 1500 female patients from southern Peru, Ecuador, Argentina, Colombia, and Venezuela. The altitude range in the Andes area where the patients come from is between 2060 and 6960 m above sea level. Information was collected from the 1500 patients which showed the average waist circumference measurement of 87.11 cm, the average chest measurement of 107.17 cm, the distance between the last palpable rib and the iliac crest averaged 7.8 cm, and in all cases was <10 cm, and finally, the average distance between the iliac crest and the trochanter, which we define as “high crest,” was 14.3 cm. Based on the results we attained from our 1500 patients, when similar studies are performed on other non-Andean groups, comparisons can be made which could help plastic surgeons in determining the appropriate abdominal contouring procedure.

**Level of Evidence: 5 (Diagnostic):**

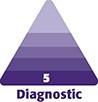

Humans have great biological plasticity, and although cultural adaptations are an important part of their evolutionary success, biological adaptations such as body shape also play a key role.

Sheldon's classification is currently recognized as including endomorphic, ectomorphic, and mesomorphic bodies, which have different physical characteristics depending on the distribution of fat and muscle.

This study focuses on Andean patients, described as inhabitants of the Andes and higher altitudes of Peru, Argentina, Chile, Bolivia, Ecuador, Colombia, and western Venezuela, in whom anatomical differences have been found to be taken into account.^1^ There is a significant relationship between climatic variables and thoracic size: the higher the altitude, and therefore the colder, the greater the size of the thoracic cage in terms of width and depth. Chronic exposure to high altitude is compensated for by a reduction in the partial pressure of oxygen (APO2), with anatomical and morphological/functional changes.^2^

In view of these environmental characteristics, it has been observed that, because of adaptation, there is a change in the arrangement of the bones in people from the Andean zone. And from professional practice, it has been observed that Andean patients undergoing plastic surgery wish to achieve a reduction in the size of the chest to have a more stylized trunk contour.

The authors of this study, based on a theoretical review and professional experience, propose a definition of the Andean body and its characteristics.

## METHODS

The present study was conducted in accordance with the guidelines of the Declaration of Helsinki, and the research protocol was approved by the institutional research and ethics board of the participating clinics.

This is a multicenter study, conducted in private clinics in Lima-Peru, Ecuador, Argentina, Colombia, and Venezuela between May 2022 and May 2024. Patients enrolled in the study underwent medical evaluation for rib reshaping and most underwent body contouring procedures, specifically abdominoplasty and liposuction. The sample consisted of 1500 female patients aged 18 to 40 years, from the Andean area of Peru, Ecuador, Argentina, Colombia, and Venezuela. The exclusion criteria were patients with a history of surgery (in plastic surgery or other specialties), a surgical risk score ≥ Goldman risk index Class II, a BMI >28 kg/m^2^.

This study has been divided into 2 parts.

### Collection of Background Information

This stage seeks to collect sociodemographic information and conditions of the place of birth, considering environmental/climatic characteristics. Also, characteristics related to physical appearance, such as weight, height, and BMI.

### Medical Assessment

**Figure 1.**
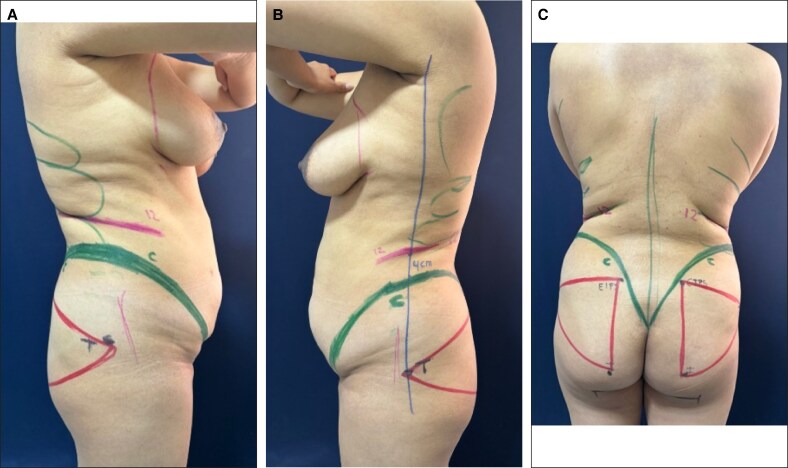
A 39-year-old female (height 1.68 m, weight 68 kg with a BMI 24.09 kg/m^2^). (A) Body marking—right profile. (B) Body marking—left profile. (C) Body marking—back.

The following criteria were considered for taking measurements and collecting data on the patients (Figure 1A-C):

- Waist: measurement in centimeters of the circumferential length between the thorax and pelvis, normally located at the midpoint of Rib 12 and the iliac crest;
- Chest: measurement in centimeters at the level of the greatest thoracic circumference, normally located at the level of the 10th rib;
- Distance in centimeters between the last palpable rib and the iliac crest;
- Distance in centimeters between the iliac crest and the trochanter.

The sociodemographic data and the medical assessment of each patient were recorded in an Excel database for statistical analysis that was performed in SPSS v.29 (IBM, Armonk, NY), using descriptive analysis and Friedman's nonparametric test.

## RESULTS

The present study was conducted in 1500 patients, women aged 18 to 40 years with no comorbidities, a mean age of 32.8 years, an average weight of 55.42 kg, an average height of 1.53 m, and an average BMI of 21.94 (Table 1).

**Table 1.** Sociodemographic Data of the Sample

Variable	Minimum	Maximum	Mean	SD
Age	18	40	32,8	3.58
Weight	45	66	55.42	4.21
Height (cm)	150	160	153	3.09
BMI	18.4	26.3	21.94	1.82
Comorbidity	—	—	None was reported	—

SD, standard deviation.

Of the total patients evaluated, 96% were seeking rib remodeling surgery, which is why the variables waist, chest, iliac crest–trochanter, and iliac crest–last palpable rib measurements were established for the description of the Andean body.

The altitude range in the Andes area where the patients come from is between 2060 and 6960 m above sea level. Of the total number of patients evaluated, 40% (600 cases) were from southern Peru, from the cities of Arequipa, Cusco, Huaraz, and Puno. These cities are located between 2355 and 3827 m above sea level. Temperatures in these cities can range between 8°C and 20°C in cooler seasons; however, they can reach average minimum temperatures below freezing.

Likewise, 20% (300 cases) come from Ecuador, from cities, such as Puyango, Riobamba, Licto, Guano, and Loja, which are located between 2060 and 3029 m above sea level.

Also, 16% (240 cases) are from Argentina, coming from areas ranging between 3000 and 6960 m above sea level. Fourteen percent (210 cases) come from Colombia, and finally, 10% (150 cases) come from Venezuela, from areas up to 4980 m above sea level.

**Figure 2.**
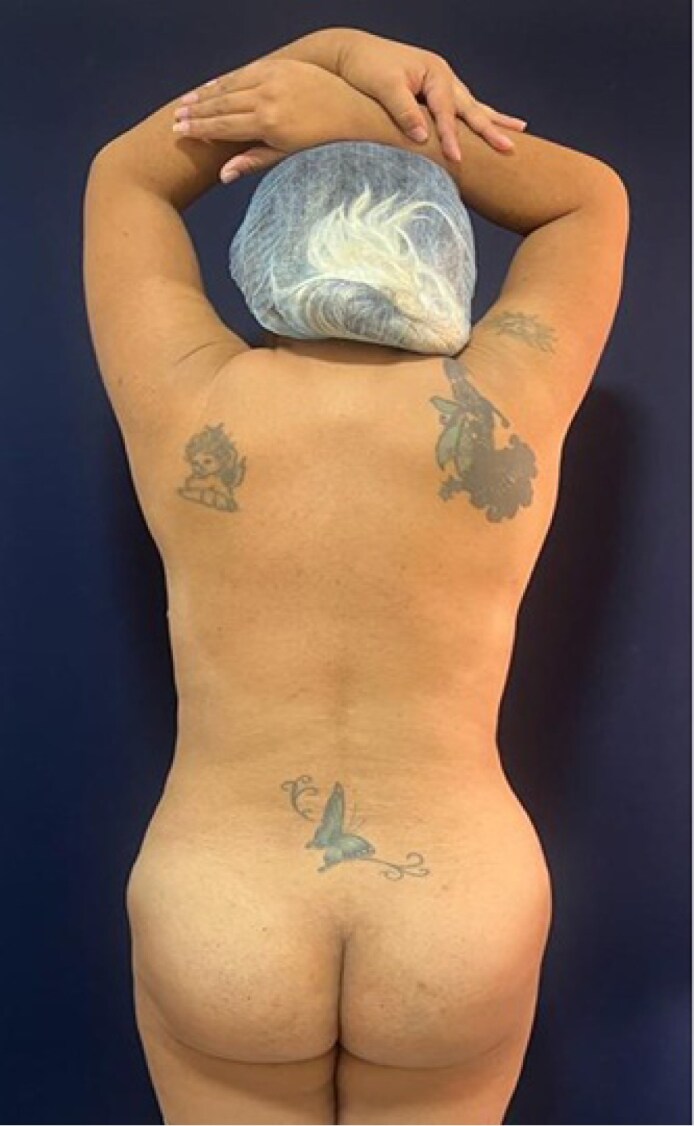
Thorax with greater width and depth of a 29-year-old female patient (height 1.66 m, weight 66 kg with a BMI 23.95 kg/m^2^).

**Figure 3.**
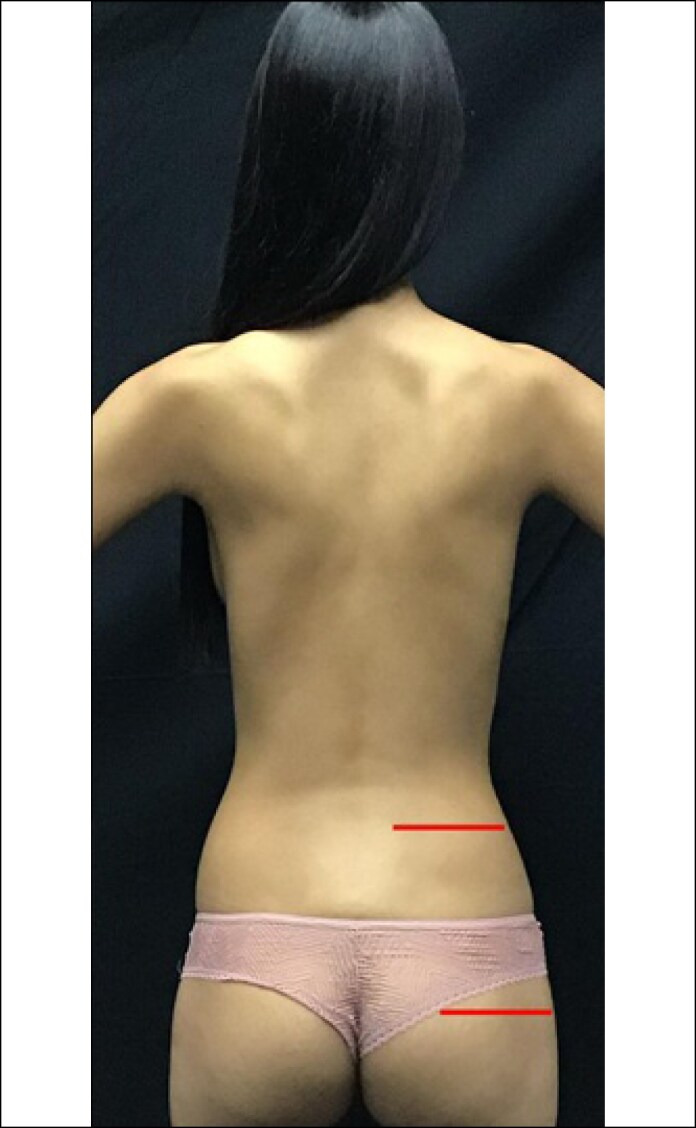
High crest: distance between the iliac crest and the trochanter >13 cm of a 23-year-old female patient (height 1.7, weight 54 kg with a BMI 18.69 kg/m^2^).

In terms of physical characteristics, information was collected from the 1500 patients and the following common characteristics were found (Table 2):

- Waist: the waist circumference measurement defined as the distance between the iliac crest and the last palpable rib presents an average of 87.11 cm.
- Chest: it has been identified that they have an average chest measurement of 107.17 cm (Figure 2).
- The distance in centimeters between the last palpable rib and the iliac crest averaged 7.8 cm and in all cases was <10 cm.
- Finally, the distance in centimeters between the iliac crest and the trochanter, presents an average of 14.3 cm, which for the purpose of learning we will define in this research as “high crest” (Figure 3).

**Table 2.** Measurements—Waist, Thorax, and Iliac Crest

Variable	Median	IR	*χ* ^2^	*P*-value
Waist measurements (cm)	87.11	(63-117)	90	.00001
Thorax measurements (cm)	107.17	(81-115)	90	.00001
Distance between the last palpable rib and the iliac crest (cm)	7.8	(6-10)	90	.00001
Distance between the iliac crest and trochanter (cm)	15.23	(13-18)	90	.00001

Friedman's nonparametric test. IR, interquartile range.

## DISCUSSION

Advances in plastic surgery not only require innovation in techniques, but it is also advisable to make comprehensive proposals. An understanding of the anatomy of the body from a cultural perspective facilitates specialized surgical interventions that are adapted to the needs according to the physical characteristics of the body.^2,3^

The theoretical review allows us to observe physical alterations at the body level that have allowed the morphological adaptation of bodies to physical conditions, such as location, altitude, and climate.^1,4^ Considering these differences helps to clarify the most appropriate surgical techniques. For a long time, surgical techniques have been based on the optimal waist/hip ratio being 0.7,^5^ and from this study, rather than questioning, we seek to provide new references for a specific population, and we emphasize personalizing plastic surgery according to the characteristics of the patients.

This study, based on the measurements taken, proposes to describe the physical characteristics present in people from the Andean areas of Peru, Ecuador, Argentina, Colombia, and Venezuela, establishing a range of 81 to 115 cm for the width of the rib cage, a range of 63 to 117 cm for the waist, with a distance between the iliac crest and the trochanter >13 cm (high crest) and an average distance between the last palpable rib and the iliac crest of <10 cm.

Currently, there are rib remodeling techniques, such as RibXcar^6^ (rib remodeling without incisions, with monocortical fracture guided by ultrasound), a technique performed through a skin puncture that leaves no scar and is minimally invasive, with favorable aesthetic results for patients. This technique is designed to reduce the size of the floating ribs, also known as the lower ribs. These ribs are flexible and can therefore be modified to help shape the desired waistline. This is achieved by controlled fracture of the external cortex through the stitch maneuver, which involves sustained punctures transverse to the direction of the ribs, thus reducing the bony cohesive force, or rib removal, which achieves a narrower waist and with favorable and satisfactory aesthetic results for patients. However, considering the physical characteristics of the Andean body, it is relevant to propose surgical techniques adapted to this type of body, such as waist reduction through the transformation of false ribs into floating ribs,^7^ a technique that improves the final result of the treatment of false ribs through a rib treatment scheme that, when combined with RibXcar, shows a greater reduction in waist circumference and more efficient rib angulation.

Regarding the limitations of this study, we believe that although the sample size is quite large, the contribution of this study is only theoretical, and the results cannot be generalized. However, it is an important basis for future research and for proposing surgical interventions based on the characteristics found.

We also believe that by exploring this topic, we can define a body type with a perspective that considers the environment, the area of origin and its influence on physical characteristics. It also proposes new concepts such as the definition of high crest.

We believe that to better understand and intervene with our patients, it is essential to understand bodies from a cultural perspective that responds to their particularities.

## CONCLUSIONS

The physical characteristics of Andean people are defined by a waist measurement between 63 and 117 cm, a chest measurement between 81 and 115 cm, a distance between the last palpable rib and the iliac crest between 6 and 10 cm, and a distance between the iliac crest and the trochanter between 13 and 18 cm.

Considering the data we observed and reported, we believe that they allow us to understand the characteristics found in this sample and set an important precedent to be taken as a reference for future research.
